# Identification of key genes and imbalance of immune cell infiltration in immunoglobulin A associated vasculitis nephritis by integrated bioinformatic analysis

**DOI:** 10.3389/fimmu.2023.1087293

**Published:** 2023-03-21

**Authors:** Xianxian Jia, Hua Zhu, Qinglian Jiang, Jia Gu, Shihan Yu, Xuyang Chi, Rui Wang, Yu Shan, Hong Jiang, Xiaoxue Ma

**Affiliations:** ^1^ Department of Pediatrics, The First Hospital of China Medical University, Shenyang, China; ^2^ Department of General Pediatrics, Zhongshan City People’s Hospital, Guangzhou, China; ^3^ First Department of Internal Medicine, School of Medicine, University of Occupational and Environmental Health, Kitakyushu, Japan; ^4^ Department of Microbiology & Immunology and Pediatrics, Dalhousie University, Halifax, NS, Canada

**Keywords:** immunoglobulin A associated vasculitis nephritis, bioinformatics analysis, differentially expressed genes, hub genes, immune infiltration

## Abstract

**Background:**

IgAV, the most common systemic vasculitis in childhood, is an immunoglobulin A-associated immune complex-mediated disease and its underlying molecular mechanisms are not fully understood. This study attempted to identify differentially expressed genes (DEGs) and find dysregulated immune cell types in IgAV to find the underlying pathogenesis for IgAVN.

**Methods:**

GSE102114 datasets were obtained from the Gene Expression Omnibus (GEO) database to identify DEGs. Then, the protein-protein interaction (PPI) network of the DEGs was constructed using the STRING database. And key hub genes were identified by cytoHubba plug-in, performed functional enrichment analyses and followed by verification using PCR based on patient samples. Finally, the abundance of 24 immune cells were detected by Immune Cell Abundance Identifier (ImmuCellAI) to estimate the proportions and dysregulation of immune cell types within IgAVN.

**Result:**

A total of 4200 DEGs were screened in IgAVN patients compared to Health Donor, including 2004 upregulated and 2196 downregulated genes. Of the top 10 hub genes from PPI network, *STAT1, TLR4, PTEN, UBB, HSPA8, ATP5B, UBA52*, and *CDC42* were verified significantly upregulated in more patients. Enrichment analyses indicated that hub genes were primarily enriched in Toll-like receptor (TLR) signaling pathway, nucleotide oligomerization domain (NOD)-like receptor signaling pathway, and Th17 signaling pathways. Moreover, we found a diversity of immune cells in IgAVN, consisting mainly of T cells. Finally, this study suggests that the overdifferentiation of Th2 cells, Th17 cells and Tfh cells may be involved in the occurrence and development of IgAVN.

**Conclusion:**

We screened out the key genes, pathways and maladjusted immune cells and associated with the pathogenesis of IgAVN. The unique characteristics of IgAV-infiltrating immune cell subsets were confirmed, providing new insights for future molecular targeted therapy and a direction for immunological research on IgAVN.

## 1 Introduction

Immunoglobulin A associated vasculitis (IgAV), also known as Henoch-Schonlein purpura, is a common systemic vascular inflammatory disease that chiefly manifests as skin purpura, arthritis, abdominal pain, gastrointestinal bleeding, and kidney involvement (1, 2), with an annual incidence of 13-20/100,000 (3). It is characterized by immunoglobulin A-dominant immune deposition in small blood vessels, and the excessive production of various inflammatory molecules. Renal involvement occurs in up to 40% of cases, and approximately 3% of cases with IgAV nephritis (IgAVN) progress to end-stage renal disease (1). Thus, the severity of kidney involvement often directly affects the course and prognosis of the disease. Despite major research progress, the underlying molecular mechanisms of IgAVN remain elusive. Therefore, it is crucial to study the pathogenesis of IgAVN and identify the underlying molecular markers relevant to the personalized therapy of IgAVN.

Recently, rapidly evolving bioinformatic and functional analysis play an important role to define the fundamental mechanisms of disease pathogenesis. By integrative analysis of transcriptomics and proteomics, Xie et al. identified a cluster of serums mRNAs and proteins and four pathways including JAK-STAT, mTOR, SWI/SNF and Wnt closely related to IgAVN progression (4). By bioinformatic study of proteins identified by iTRAQ, Gao et al. revealed that differential genes were mainly enriched in lipid metabolism and adhesion connection pathways, among which CDC42 and CTNNB1 were potential candidates involved in the pathogenesis of IgAVN (5). Based on urinary proteomics analysis, Fang et al. assessed the development of IgAVN in children and discovered the signaling pathways involved, such as cell adhesion and PI3K-AKT signaling (6). These studies provided new insights into the pathogenesis of IgAVN. Indeed, IgAV is a systemic vasculitis, which involves systemic dysregulation of immunity. Abnormal expression of immune factors and imbalance of immune cell differentiation in general circulation may play crucial roles in the pathogenesis of IgAVN (7). However, up to now, the biomarkers of IgAVN immunological characteristics are still lacking. Therefore, it is necessary to identify key pathways and immune cells in vasculitis, and to assess the significance of the immune microenvironment in the pathogenesis of IgAVN.

In the present study, through bioinformatics analysis, validation of hub genes expression and assessment of peripheral blood lymphocyte phenotype from patients with IgAVN, we aimed to investigate the potential immune modulatory mechanisms of IgAVN to construct novel underlying candidate biomarkers for the effective prediction of therapeutic targets in the future.

## 2 Materials and methods

### 2.1 Data collection and identification of differentially expressed genes (DEGs)

The GSE102114 contained 10 peripheral blood samples including four healthy donors (HD) and six IgAVN patients submitted by Pang et al. (8) was acquired from the Gene Expression Omnibus (GEO) dataset (http://www.ncbi.nlm.nih.gov/geo/) (9, 10). The raw count data of GSE102114 was generated using the GPL11154 platform. As for the data processing, the raw count data was transformed into counts per million (cpm) data using the R package “edgeR” firstly (11–13). The “filterByExpr” function in the “edgeR” package was used to automatically select and exclude the genes with low-expression. Then, the “voom” function of the “limma” package was utilized to normalize the logCPM expression value of the total samples based on the quantile normalization method (14). Principal component analysis (PCA) and hierarchical cluster analysis were used to reflect the similarity and difference among different samples. Subsequently, the differentially expressed genes between IgAVN patients and HD were calculated using the edgeR package in R. The significantly differentially expressed genes (DEGs) were selected with the criteria of adjusted *p*-value <0.05 and |Log_2_Fold change| >2. Thereafter, ggplot2 and heatmap packages in R were used to draw the volcano and heatmap to visualize these DEGs.

### 2.2 Construction of the protein-protein interaction (PPI) network

Because proteins rarely work alone, it is necessary to investigate the interactions among proteins. To identify more important protein interactions, we selected DEGs with adjusted *p*-value <0.05 and |Log_2_Fold change| >2. In addition, PPI network of the DEGs was performed with a confidence score of >0.7 according to the Search Tool for the Retrieval of Interacting Genes (STRING, version 11.0, http://string-db.org) database (15, 16), a database of known and predicted PPIs according to the sources originating from computational predictions, high-throughput experiments, automated text mining, and co-expression networks (17). The STRING analysis results were entered into Cytoscape software (version 3.8.0) to construct the molecular interaction network diagrams.

### 2.3 Identification of hub genes

By computing the scores of the two ranked methods separately, including Betweenness and Bottleneck, the top 25 genes for each method were selected. A venn diagram was constructed to show the common hub genes according to these two approaches. Finally, the function of hub genes was analyzed using the clusterProfiler package in R.

### 2.4 Signaling pathway enrichment analysis

The Gene ontology (GO) analysis is usually applied to annotate genes and their products, while the Kyoto Encyclopedia of Genes and Genomes (KEGG) pathway database is used to identify functional and metabolic pathways. To determine the potential biofunctions of the hub genes, GO and KEGG pathway enrichment analyses were performed. Functional enrichment analysis of DEGs was conducted using the R package “clusterProfiler” (version 4.6.0) (18–20). Furthermore, the Gene Set Enrichment Analysis (GSEA) based on the DEGs using KEGG subset of canonical pathways, reactome pathway gene sets, and immunologic signature gene sets was utilized to identify the potentially immunological pathways associated with the disease (21, 22).

### 2.5 Characterization of infiltrating immune cells

The R package “ImmuCellAI” was employed to evaluate the proportion of infiltrating immune cells based on default parameters and FPKM format by RNA-seq data was used as input data. ImmuCellAI is an analytical tool that predicts the abundance of twenty-four immune cell types based on gene expression data according to a gene set signature approach, containing eighteen T-cell subtypes and six other immune cells: B cells, dendritic cells (DCs), natural killer (NK) cells, monocytes, macrophages, and neutrophils (23, 24). The Wilcox test was used to assess the difference in immune cell proportions between HD and IgAVN patients and *p* value less than 0.05 was considered as significantly different. Principal component analysis (PCA) was used to reflect the similarities and differences among different samples based on immune infiltration characteristics. The correlation heatmap was plotted using the R package “corrplot” to demonstrate the correlations between 24 types of infiltrated immune cells. The “pheatmap” package in R was used to create a heatmap of the degree of infiltration of 24 immune cells in all samples. The violin plots revealed that the differential expression of 24 types of immune cells was drawn by the vioplot package in R.

### 2.6 Subjects for validation

All patient experiments were approved by the Medical Science Research Ethics Committee of the First Affiliated Hospital of China Medical University (2016-210-2). IgAVN subjects that fulfill the European League Against Rheumatism/Pediatric Rheumatology International Trials Organization/Pediatric Rheumatology European Society criteria were included (25). IgAVN patients meeting any of the following criteria were excluded from this study: (1) Suffering from other autoimmune diseases, allergic diseases, chronic inflammatory diseases, acute or chronic infectious diseases, etc (2) hormonal and/or immunosuppressive drugs are being used. The age and sex of the HD group were matched with those of the IgAVN group. Peripheral blood mononuclear cells (PBMC) were extracted with a lymphocyte separator (Cytiva Sweden AB). For PCR experiments, IgAVN patients (n=28) and HD (n=35) were collected. For flow cytometry experiments, IgAVN patients (n=20 for assay of transcription factors/n=30 for determination of surface molecular markers) and HD (n=25 for assay of transcription factors/n=30 for determination of surface molecular markers) were collected. The baseline demographics and clinical characteristics of patients with IgAVN are summarized separately in **Supplementary Tables S1, S2** and **S8**. The study protocol was reviewed and approved by the First Hospital of China Medical University.

### 2.7 Quantitative Reverse-Transcription PCR (qRT-PCR)

Total RNA was isolated from PBMC using TRIzol reagent (9108, Takara, Japan). Reverse transcription (RR047A, Takara) and amplification (RR820A, Takara) were performed on the Applied Biosystems™ Fluorescence Quantitative PCR instrument according to the instructions. All primer sequences are listed in **Supplementary Table S3**. The 2^−ΔΔCt^ method was used to determine relative expression. qRT-PCR was performed using 28/35 biological replicates. *GAPDH* mRNA was used as the normalization standard.

### 2.8 Flow cytometry

PBMCs were stained at 4°C for 30 minutes for surface staining. Before intracellular staining, PBMCs were fixed at 4°C for 50 minutes with fixation and permeabilization buffer (eBioscience, Thermo Fisher Scientific). The antibodies and isotype controls used in this study are presented in **Supplementary Table S4**. The samples were examined with a FACSAria type flow cytometer (BD, United States). Data were analyzed using FlowJo 10.0 software.

### 2.9 Statistical analysis

All analyses were performed using the R language (version 3.6.1) and relevant packages. All data are shown as mean ± standard deviation (SD). Statistical analyses were performed using GraphPad Prism (version 8.0). A two-tailed Student’s *t*-test was used to evaluate the statistical significance. Statistical significance was set at *p* < 0.05.

## 3 Results

### 3.1 Identification of differential expressed mRNA between IgAVN patients and HD

**Figure 1:**
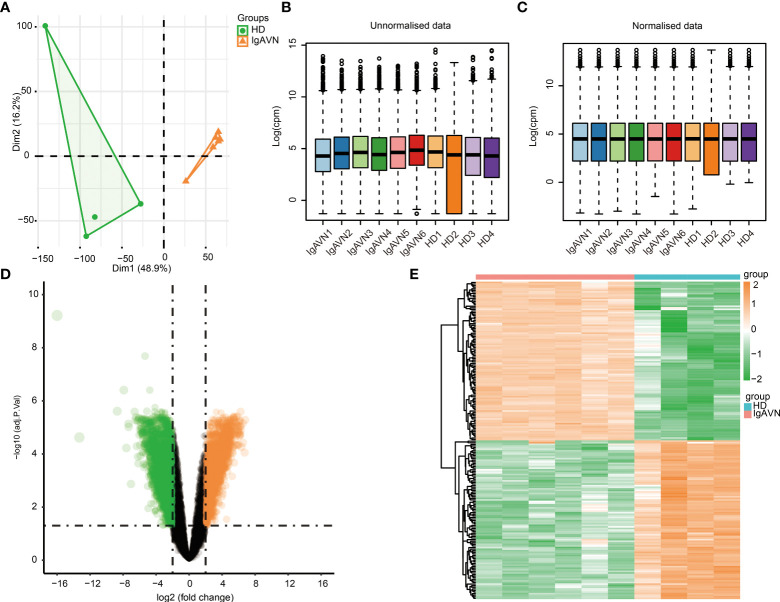
Identification of differentially expressed genes between four healthy donors (HD) and six Immunoglobulin A associated vasculitis nephritis (IgAVN) patients. **(A)** Principal component analysis (PCA) was used to cluster all the samples in the GSE102114 dataset. **(B)** The boxplot of gene expression before the normalization of GSE102114 dataset. **(C)** The boxplot of gene expression after the normalization of GSE102114 dataset. **(D)** Volcano plot used to demonstrate the differentially expressed genes. The orange points represent genes with upregulated expression screened based on log_2_fold change >2 and adjusted *p* <0.05. The green points represent the downregulation of the expression of genes screened based on log_2_fold change <-2 and adjusted *p* value <0.05. The black points represent genes with no significant difference. **(E)** Hierarchical clustering heatmap of DEGs. Orange represents higher expression and green represents lower expression.

The results after we processed the data are as follows. PCA was used to verify the biological differences among different samples (**Figure 1A**). The samples of IgAVN and HD were grouped separately, indicating a globally different expression profile. The hierarchical clustering of the samples divided the ten samples into two main clusters (**Figure S1A**). The density of gene expression for individual samples before (**Figure S1B**) and after (**Figure S1C**) filtering for low expression genes. Our results showed the expression distribution of unnormalized (**Figure 1B**) and normalized data (**Figure 1C**), in which the distribution of different samples is significantly different before normalization, but similar after normalization. The voom plot represents that experiments with large biological differences usually tend to be flat, and their variance values are stable at high expression. Before filtering for low expression genes and normalization, there will be a decreasing trend between mean and variance in the voom plot (**Figure S1D**). The voom chart shows a stable trend after data processing (**Figure S1E**). As can be seen from the figure, the variance is no longer correlated with the mean expression level. Diagrams such as PCA analysis, hierarchical clustering, and boxplots, were used to exhibit the process of data preprocessing.

A total of 4,200 DEGs were screened (|Log_2_fold change| > 2, adjusted *p* < 0.05) based on the analysis in IgAVN samples compared to HD samples, which contained 2,102 genes with upregulated and 2,098 with downregulated expression. In addition, a volcano plot of all DEGs was created using the ggplot2 package in R (**Figure 1D**). Subsequently, the expression levels of DEGs were hierarchically clustered, and the results were visualized in the form of heatmaps (**Figure 1E**). The top 20 upregulated and top 20 downregulated mRNA expressions in the IgAVN group are exhibited in **Supplementary Table S5**. These data suggest that the expression levels of mRNAs were distinguishable and variable between the two groups, implying underlying pathological changes in the IgAVN samples.

### 3.2 Identification of 20 overlapping hub genes and enriched pathways analysis

A PPI network was generated based on the STRING database to study further the underlying relationships between the proteins encoded by DEGs. After removing isolated and partially connected nodes, a complicated network of DEGs was created. Network analysis of the DEGs revealed 1,238 nodes and 15,046 edges in the PPI network (**Figure S2A**). The nodes correspond to genes, and the edges represent the links between genes. Orange represents genes with upregulated expression, and green represents genes with downregulated expression.

**Figure 2:**
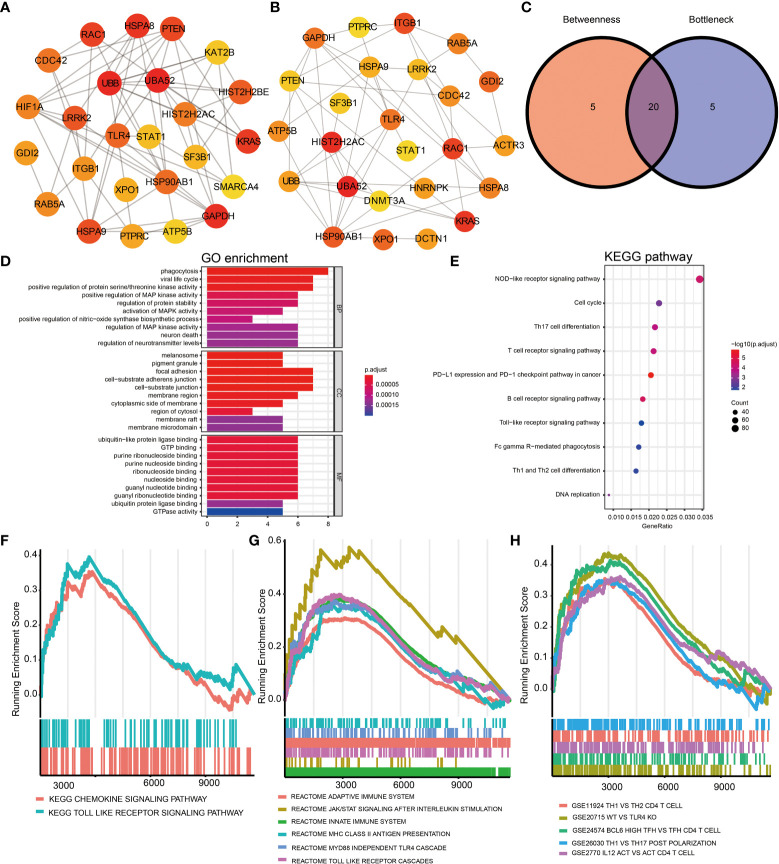
Identification of hub genes of DEGs based on the PPI network and signaling pathways enrichment using GO, KEGG, and GSEA analysis. **(A)** The top 25 hub genes obtained by Betweenness from the PPI network. **(B)** The top 25 hub genes obtained by Bottleneck. **(C)** 20 hub genes determined by intersection of the two methods. GO **(D)** and KEGG **(E)** analysis of 20 hub genes. Identification of immune-related signaling pathways using GSEA analysis based on the reference genesets including KEGG subset of canonical pathways **(F)**, reactome pathway gene sets **(G)**, and immunologic signature gene sets **(H)**. DEGs, differentially expressed genes; GO, Gene Ontology; KEGG, Kyoto Encyclopedia of Genes and Genomes; BP, biological process; CC, cellular component; MF, molecular function; GSEA, gene set enrichment analysis; Protein-protein interaction, PPI.

The top 25 genes were screened out by the two classification approaches in cytoHubba (**Figures 2A, B**). Finally, the venn diagram identified 20 overlapping hub genes according to these two approaches (**Figure 2C** and **Supplementary Table S6**), such as *ATP5B, STAT1, HSPA8, UBB, PTEN, UBA52, ITGB1, TLR4, HIST2H2AC*, and *CDC42*. The enriched GO terms and KEGG pathways analyzed *via* the R package are displayed in **Figures 2D, E**. Hub genes participating in GO terms included activation of mitogen-activated protein kinase (MAPK) activity, regulation of MAPK activity, positive regulation of MAPK activity, and regulation of protein stability. The most significant KEGG pathways of hub genes were the Toll-like receptor (TLR) signaling pathway, nucleotide oligomerization domain (NOD)-like receptor signaling pathway, T helper (Th) 17 cell differentiation. Finally, the GSEA analysis were utilized to further identify immunological signaling pathways based on the reference genesets including KEGG subset of canonical pathways (**Figure 2F**), reactome pathway (**Figure 2G**), and immunologic signature (**Figure 2H**). We identified that Th1, Th2, Th17, Tfh, JAK/STAT signaling pathway, TLR signaling pathway were significantly enriched, contributing to elucidate the potential mechanisms behind the occurrence of IgAVN.

### 3.3 Validation of the expression of top 10 of hub genes

**Figure 3:**
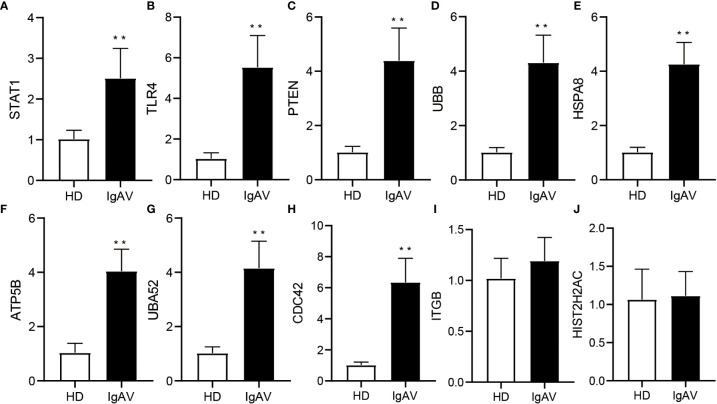
Validation of DE hub genes expression using qRT-PCR in IgAVN patients and HD from the pediatric ward. **(A-J)** Changes in mRNA expression were confirmed for select top 10 of hub genes in the IgAVN and HD groups by qRT-PCR. Data are given as the mean ± SD (n = 28/35); **P<0.01.

To check the accuracy and credibility of the data and to lay the foundation for further research, top 10 of hub genes were selected for qRT-PCR analysis. Peripheral blood samples from IgAVN patients (n=28) and HD (n=35) were collected for validation. We found that eight out of 10 hub genes including *STAT1, TLR4, PTEN, UBB, HSPA8, ATP5B, UBA52*, and *CDC42* were upregulated, which is consistent with previous sequencing analyses (**Figures 3A–J**). Therefore, the data of bioinformatic analysis were almost consistent with the expression levels verified by qRT-PCR.

### 3.4 Higher immune cell infiltration in IgAVN compared with HD

**Figure 4:**
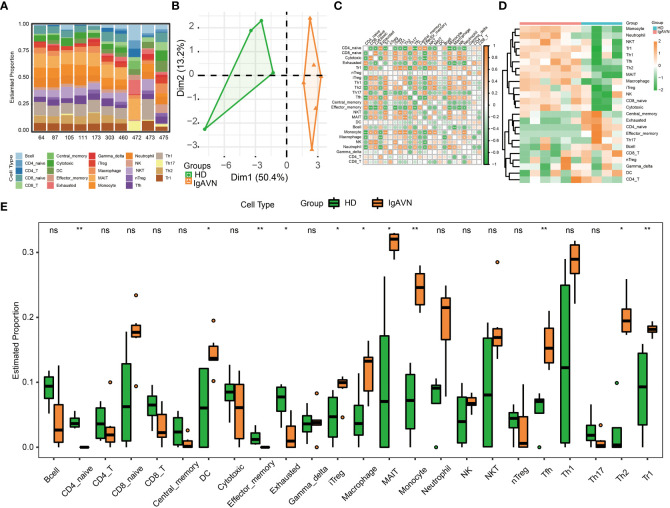
Profiles of Immune Cell Infiltration in Immunoglobulin A associated vasculitis nephritis (IgAVN) and healthy donors (HD) Based on Gene Expression Data. **(A)** Bar plot of infiltration level of 24 kinds of immune cell in HD and IgAVN samples. **(B)** Principal component analysis (PCA) analysis of HD and IgAVN groups based on the immune infiltration level of 24 kinds of immune cells. **(C)** The correlation matrix of immune cell proportions between HD and IgAVN patients. **(D)** Heatmap of the 24 kinds of immune cell proportions in the two groups. **(E)** Comparisons of the proportions of 24 immune cell types between the HD and IgAVN group. The HD group is marked in green, and the IgAVN group is marked in orange. Tc, cytotoxic T cell; Tex, exhausted T cell; Tcm, central memory T cell; Tem, effector memory T cell; Tgd, gamma delta T cell. ns, not significant *P <0.05; **P <0.01.

Next, we intended to gain some insight into the immune cell infiltration nature of IgAVN peripheral blood samples using ImmuCellAI analysis. A total of 24 subpopulations of immune cells and its corresponding percentage of each samples are shown in **Figure 4A**. We discovered that T cells, especially Th2, mucosal-associated invariant T (MAIT) cells, Th1, Tfh, Th17 and exhausted T cell, dominated the peripheral blood. Because of the remarkable change in immune cells in IgAVN, we deduced that changes in the fractions of immune cells might be applied to show individual differences. The abundance of immune cells in the 10 samples revealed striking group-bias clustering in the PCA map (**Figure 4B**), indicating vast internal heterogeneity in the immune spectrum is exist between IgAVN and HD samples. Additionally, we investigated the internal correlations between different immune cell types which might assist in exploring immune cell-cell interactions in the future (**Figure 4C**). According to the hierarchical clustering of 24 immune cell subgroups (**Figure 4D**), we discovered some modules where specific styles of immune cells had a similar expression, which showed a cooperative effect in IgAVN. Compared with HD, IgAVN samples had higher infiltration scores, indicating a greater abundance of immune cells and altered fractions of immune cell subgroups (**Figure 4E** and **Supplementary Table S7**). Comparing the immune cell fractions of peripheral blood samples, 13 of the 24 immune cells were significantly different. Higher proportions of Th2, monocytes, mucosal associated invariant T (MAIT), macrophages, induced T regulatory (iTreg), T follicular helper (Tfh), and T regulatory 1 (Tr1) were identified in IgAVN. In comparison, lower proportions of naive CD4^+^T cells and effector memory were identified in IgAVN. In addtion, we performed correlation analysis between 10 hub genes and subtypes of CD4^+^T cell (**Figure S3**). We found a common correlation between T helped cell subsets and hub genes.

### 3.5 Validation of phenotype frequency of CD4^+^T cell subsets

**Figure 5:**
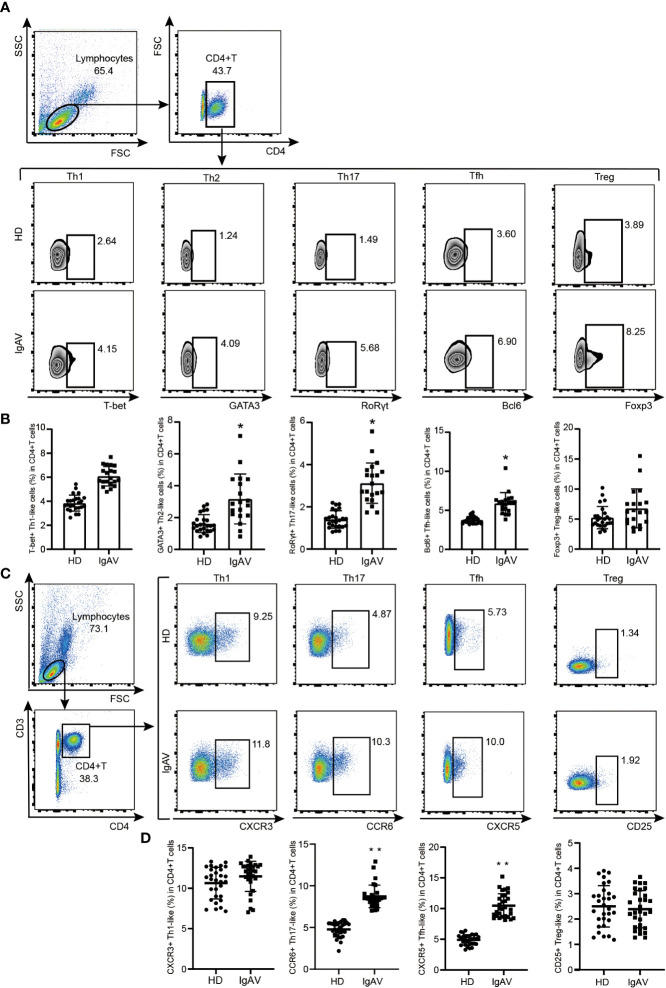
Validation of phenotype of CD4^+^T cell subsets in the IgAVN patients and HD from the pediatric ward. **(A, B)** Changes in the proportions of Th1, Th2, Th17, Tfh and Treg in the IgAVN and HD groups by flow cytometry. Data are given as the mean ± SD (n = 20/25) **(C, D)** Changes in the proportions of Th1, Th17, Tfh and Treg in the IgAVN and HD groups by flow cytometry. Data are given as the mean ± SD (n = 30); *P <0.05; **P <0.01.

Since both hub genes enrichment analysis and ImmuCellAI analysis displayed that there was significant difference in the development of Th cells between IgAVN and HD, a validation of phenotype frequency of CD4^+^T cell subsets was performed using flow cytometry. T box factor (T-bet), GATA binding protein 3 (GATA3), retineic-acid-receptor-related orphan nuclear receptor gamma (RORγt), B-cell lymphoma 6 (Bcl-6) and forkhead box protein 3 (Foxp3) are respectively the major transcription factor of Th1, Th2, Th17, Tfh, and T regulatory (Treg) cells. Meanwhile, chemokine C-X-C-Motif Receptor 3 (CXCR3), CC chemokine receptor 6 (CCR6), chemokine C-X-C-Motif Receptor 5 (CXCR5), CD25 is a molecular marker of Th1, Th17, Tfh and Treg cell surface, respectively (26). We found that frequencies of CD4^+^RORγt^+^Th17/CD4^+^CCR6^+^Th17, CD4^+^GATA3^+^Th2, and CD4^+^Bcl-6^+^Tfh/CD4^+^CXCR5^+^Tfh cells were significantly increased in IgAVN patients compared with HD (**Figures 5A–D**).

## 4 Discussion

Although the pathogenesis of IgAVN remain unclear, it may involve the disruption of both cellular and humoral immunity, and the release of various cytokines and inflammatory mediators (27, 28). In our GO and KEGG enrichment analysis, hub genes were enriched in both the innate immune pathways (TLR signaling, NOD-like receptor signaling, and MAPK signaling) and the adaptive immune pathways (Th17 cell differentiation). In ImmuCellAI analysis, compared with HD, IgAVN samples had higher infiltration scores, implicating the altered abundance of innate immune cells (Macrophage, Monocyte, and Neutrophil) and adaptive immune cells (DC, B cell and multiple T cell subgroups).

It was shown by epidemiological statistics that infections are a major precipitating factor for IgAV. Almost 80% of children who have IgAV had an infection at the time of diagnosis (29). During prophase infection, pathogens activate innate immune response that leads to the production of immune-effector molecules. Our analysis showed that the most significant KEGG pathways of hub genes were TLR signaling pathway. TLRs are a group of important pattern recognition receptors participated in the innate immune system that can identify pathogen-associated molecular patterns to activate cell signaling mechanisms (30–33). Studies showed that TLR2 activation was correlated with the increased immunoproteasome switch in IgAV (34–36). TLR4 overproduction in IgAV patients provokes renal impairment (35, 36). In our study, *TLR4* was one of overlapping hub genes. Validation of experiments also indicated that the transcription of *TLR4* from IgAVN patients was significantly higher than HD. Therefore, we suggest that immune dysregulation mediated by TLR4 signaling is involved in the pathogenesis of IgAVN.

Besides innate immune, T-B cell-mediated adaptive immunity may play a crucial role in the pathogenesis of IgAV (37). Activation of TLR4 can promote the production of type I interferons (IFN-I), which is considered as the first step in the development of adaptive immunity from innate immunity (38). In the canonical pathway of IFN-I signalling mainly activated signal transducer and activator of transcription 1 (STAT1) (39). Interestingly, *STAT1* was also contained in our overlapping hub genes and verified high expressed in IgAVN patients. Our previous research suggested that activated STAT1 directly induced the expression of *BCL6*, thus being considered as a key transcript factor for the differentiation of Tfh cells (40). Consistent with our inference, imbalance of T helper cell subgroups including Tfh cells was indeed observed in IgAVN. In ImmuCellAI analysis, the fractions of immune cell subgroups in Th2 and Tfh cells was increased in IgAVN. Validation of phenotypes of CD4^+^T cell using flow cytometry also indicated that proportions of CD4^+^GATA3^+^Th2 and CD4^+^Bcl-6^+^Tfh/CD4^+^CXCR5^+^Tfh cells were higher in IgAVN patients than HD. Increasing of Tfh cells may promote the synthesis of IgA and is closely related to the progression of IgAV (41). Th2 cell hyperactivity can increase immunoglobulin synthesis and release in patients with IgAV (42–45). It is noteworthy that since Tfh cell developed through Th17-source leads to isotype switching of B cell to induce IgA production (26), the raise of Th17 cell frequency in peripheral blood and serum IL-17 level may promote the occurrence of vascular inflammation to a certain extent in patients with IgAV (46). In our study, the pathway of Th17 cell differentiation was enriched in KEGG analysis. Although an increasing of Th17 cells in IgAV patients was not indicated in ImmuCellAI analysis, the expansion of CD4^+^RORγt^+^Th17/ CD4^+^CCR6^+^Th17 cells in patients with IgAV were observed in flow cytometry investigation. Taken together, our study suggests that the over-differentiation of T helper cells, including Tfh cells, Th2 cells and Th17 cells, may be involved in the occurrence and development of IgAVN.

In addition, Treg cells can regulate the immune system, maintain tolerance to autoantigens, and prevent autoimmune diseases (47). Previous studies proved that the insufficient immunosuppression caused by the reduction of Treg cells is one of the causes of IgAV (34). However, no significant difference in the number of Treg cells in peripheral blood between IgAVN patients and HD was found in our flow cytometry investigation. In ImmuCellAI analysis, it is iTreg, but not nature Treg (nTreg), significantly increased in patients with IgAV, which indicates that environmental factors can mediate the occurrence of IgAV by inducing immune cell differentiation disorder. However, because the source of data set and the number of samples are very limited, we cannot conduct comprehensive analysis on multiple data sets. The mechanisms of T helper cells and Treg cells participating in the pathogenesis of IgAV still need further study.

In summary, a series of bioinformatics analysis methods were used to identify 4200 DEGs and 20 hub genes that may participate in IgAV progression. Additionally, we determined the immune cell composition of patients with IgAVN and identified maladjusted immune cells. However, further studies are needed to elucidate the in-depth molecular mechanisms and biological functions of these genes in IgAVN. These data may offer helpful information and direction into potential biomarkers and biological mechanisms of IgAVN.

## Data availability statement

The original contributions presented in the study are included in the article/**Supplementary Material**, Further inquiries can be directed to the corresponding author.

## Ethics statement

The studies involving human participants were reviewed and approved by all patient experiments were approved by the Medical Science Research Ethics Committee of the First Affiliated Hospital of China Medical University (2016-210-2). Written informed consent to participate in this study was provided by the participants’ legal guardian/next of kin.

## Author contributions

The study was designed by XM, XJ and HZ. XJ and HZ conducted research. QJ, JG, SY, XC, RW, HJ and YS helped analyze the data. The manuscript wrote and revised by XJ and HZ. All authors contributed to the article and approved the submitted version.

## Supplementary material

Supplementary Figure 1 Removing low expressed genes and normalization of gene expression for the GSE102114 dataset. **(A)** The hierarchical clustering of the samples from GSE102114 dataset. **(B)** The density plot of logCounts per Million (CPM) values for raw pre-filtered gene expression data. **(C)** The density plot of logCPM values after filtering low expressed genes. **(D)** The relationship between means and variances of each gene before voom. **(E)** The relationship between means and variances of each gene after voom.

Supplementary Figure 2 Construction of the protein-protein interaction (PPI) network **(A)** The PPI network of DEGs, according to the STRING online database, was constructed by Cytoscape.

Supplementary Figure 3 Correlation analysis between 10 hub genes and subtypes of CD4^+^ T cell. The orange color represent positive correlation. The green color represent negative correlation. The size of the circle represent the degree of correlation ranging from 1 to -1. **P <*0.05, ***P <*0.01, ****P <*0.001.
