# Supplementary material for: Identification of key genes and imbalance of immune cell infiltration in immunoglobulin A associated vasculitis nephritis by integrated bioinformatic analysis
Source: Front Immunol. 2023 Mar 21;14:1087293. doi: 10.3389/fimmu.2023.1087293 (PMC10070996; doi:10.3389/fimmu.2023.1087293)
Supplement: Supplementary file 1 [file Table_1.docx]

**Supplementary Table S1**

Baseline demographics and clinical characteristics of patients for PCR validation

| Characteristics | IgAVN  (n=28) | HD  (n=35) | *p* value |
| --- | --- | --- | --- |
| Age (year) | 10.1±2.9 | 8.3±2.9 | 0.307 |
| Females, n (%) | 15(55.57) | 18(51.42) | 0.868 |
| IgA (g/L) | 2.6±0.9 |  |  |
| IgG (g/L) | 11.0±2.0 |  |  |
| IgM (g/L) | 1.1±0.4 |  |  |
| CRP (mg/L) | 6.75±10.7 |  |  |
| ESR (mmol/L) | 16.4±12.2 |  |  |

**Supplementary Table S2**

Baseline demographics and clinical characteristics of patients for flow cytometry validation

| Characteristics | IgAVN  (n=20) | HD  (n=25) | *p* value | |
| --- | --- | --- | --- | --- |
| Age (year) | 10.4±3.8 | 8.1±2.4 | | 0.069 |
| Females, n (%) | 12(60) | 15(60) | | 0.9 |
| IgA (g/L) | 2.8±1.1 |  | |  |
| IgG (g/L) | 10.9±1.3 |  | |  |
| IgM (g/L) | 1.1±0.4 |  | |  |
| CRP (mg/L) | 6.3±10.0 |  | |  |
| ESR (mmol/L) | 19.5±15.5 |  | |  |

**Supplementary Table S3**

Primers used in the Study

| Primers |  | sequence |
| --- | --- | --- |
| *STAT1* | *forward:* | *ATGCTGGCACCAGAACGAATGAG;* |
|  | *reverse:* | *TCACCACAACGGGCAGAGAGG* |
| *TLR4* | *forward:* | *GCTCTTGGTGGAAGTTGAACGAATG;* |
|  | *reverse:* | *CAAGCACACTGAGGACCGACAC* |
| *HSPA8* | *forward:* | *TCCCTTGGTATTGAAACTGCTGGTG;* |
|  | *reverse:* | *GTCTGTGTCTGCTTGGTAGGAATGG* |
| *ATP5B* | *forward:* | *GTTGGCAGTGAGCATTACGATGTTG;* |
|  | *reverse:* | *GCACGGGACACGGTCAACTTG* |
| *UBB* | *forward:* | *TCCTGCGTCTGAGAGGTGGTATG;* |
|  | *reverse:* | *CTCTGCTGGTCGGGAGGGATG* |
| *PTEN* | *forward:* | *TTTGAAGACCATAACCCACCACAGC;* |
|  | *reverse:* | *TCATTACACCAGTTCGTCCCTTTCC* |
| *UBA52* | *forward:* | *CTGCCGCAAGTGCTATGCTC;* |
|  | *reverse:* | *CGCAGGTTGTTGGTGTGACC* |
| *ITGB1* | *forward:* | *TGGGCTTTACGGAGGAAGTAGAGG;* |
|  | *reverse:* | *GACACTTGGGACTTTCAGGGATGC* |
| *HIST2H2AC* | *forward:* | *GCGGCTCGGGACAACAAGAAG;* |
|  | *reverse:* | *CCTGGGCGATGGTGACTTTGC* |
| *CDC42* | *forward:* | *AGGCTGTCAAGTATGTGGAGTGTTC;* |
|  | *reverse:* | *CTGCGGCTCTTCTTCGGTTCTG* |
| *GAPDH* | *forward:* | *ACAGCAACAGGGTGGTGGAC;* |
|  | *reverse:* | *TTTGAGGGTGCAGCGAACTT* |

**Supplementary Table S4**

Antibodies used in the Study

| Antigen | Clone | Company |
| --- | --- | --- |
| CD4-FITC | RPA-T4 | BD biosciences |
| Bcl-6-APC-Cy7 | K112-91 | BD biosciences |
| RORγt-Percp | Met1­Arg10 | R&D Systems |
| T-bet-PE-cy7 | eBio4B10 (4B10) | eBioscience |
| GATA3-PAC | Pro135-Ser258 | R&D Systems |
| Foxp3-PE | PCH101 | eBioscience |
| Mouse IgG1-APC | KLH | R&D Systems |
| Mouse IgG1-percp | KLH | R&D Systems |
| Mouse IgG1-PE-Cy7 | MOPC-21 | BD biosciences |
| Mouse IgG2b-PE | KLH | R&D Systems |
| Mouse IgG1-APC-Cy7 | MOPC-21 | BD biosciences |

**Supplementary Table S5**

The top 20 upregulated and top 20 downregulated mRNA expressions between IgAV and HD groups

| name |  | Log_2_fold change |  | adjusted *p* value |  | regulation |
| --- | --- | --- | --- | --- | --- | --- |
| HBA1 |  | -15.9973714 |  | 6.08E-10 |  | down |
| HBA2 |  | -13.29871479 |  | 2.39E-05 |  | down |
| AC069368.3 |  | -8.70850753 |  | 2.45E-06 |  | down |
| RP4-583P15.15 |  | -7.91483381 |  | 3.94E-07 |  | down |
| AKR1C1 |  | -7.603800478 |  | 5.94E-05 |  | down |
| PAGR1 |  | -7.35330689 |  | 7.30E-06 |  | down |
| LA16c-431H6.6 |  | -7.323631457 |  | 9.53E-06 |  | down |
| ASTN2 |  | -7.048987542 |  | 0.001350779 |  | down |
| DDC8 |  | -6.610209292 |  | 2.56E-06 |  | down |
| COL7A1 |  | -6.50724428 |  | 5.28E-06 |  | down |
| CROCC |  | -6.441825887 |  | 4.10E-06 |  | down |
| C1ORF220 |  | -6.347473995 |  | 5.10E-05 |  | down |
| SULT1A3 |  | -6.259905652 |  | 5.86E-06 |  | down |
| CTD-2583A14.10 |  | -6.212678452 |  | 0.000127614 |  | down |
| SPTBN5 |  | -6.179752069 |  | 5.28E-06 |  | down |
| C4orf32 |  | -6.151481047 |  | 0.000228251 |  | down |
| MUC20 |  | -6.088933056 |  | 4.37E-06 |  | down |
| MC1R |  | -6.064869536 |  | 4.67E-06 |  | down |
| RP11-849H4.2 |  | -6.041766413 |  | 1.91E-05 |  | down |
| CLDND2 |  | -5.983198197 |  | 1.18E-05 |  | down |
| H3F3A |  | 5.956973152 |  | 1.23E-05 |  | up |
| B2M |  | 5.960531816 |  | 6.37E-06 |  | up |
| NAMPTL |  | 6.009867194 |  | 4.41E-05 |  | up |
| CAPZA1 |  | 6.022305508 |  | 9.19E-06 |  | up |
| IFIT5 |  | 6.074790374 |  | 1.49E-05 |  | up |
| S100A12 |  | 6.080345769 |  | 1.67E-05 |  | up |
| MT-CO1 |  | 6.126151634 |  | 2.11E-05 |  | up |
| RNF13 |  | 6.147628 |  | 6.02E-06 |  | up |
| EIF3E |  | 6.149990793 |  | 5.28E-06 |  | up |
| RGS18 |  | 6.184803031 |  | 1.89E-05 |  | up |
| DCUN1D1 |  | 6.197476531 |  | 1.89E-05 |  | up |
| IFNGR1 |  | 6.205855703 |  | 9.19E-06 |  | up |
| CLC |  | 6.238587998 |  | 9.19E-06 |  | up |
| SUMO2 |  | 6.238803957 |  | 2.56E-06 |  | up |
| CREG1 |  | 6.276181394 |  | 1.28E-05 |  | up |
| USP12 |  | 6.343154001 |  | 5.48E-05 |  | up |
| RPL39 |  | 6.510510155 |  | 7.01E-06 |  | up |
| SAMD9 |  | 6.521495262 |  | 3.76E-05 |  | up |
| IL8 |  | 6.720473467 |  | 8.84E-05 |  | up |
| STIL |  | 6.780499782 |  | 5.28E-06 |  | up |

**Supplementary Table S6**

The venn diagram identified 20 overlapping hub genes

| name |  | log_2_fold change |  | adjusted *p* value |  | regulation |
| --- | --- | --- | --- | --- | --- | --- |
| GAPDH |  | 2.036550319 |  | 0.000608536 |  | up |
| HSP90AB1 |  | 2.077035435 |  | 0.002078064 |  | up |
| KRAS |  | 2.294738661 |  | 0.000999174 |  | up |
| HSPA9 |  | 2.379058651 |  | 0.002959277 |  | up |
| XPO1 |  | 2.446788656 |  | 1.26331E-05 |  | up |
| RAB5A |  | 2.465707576 |  | 0.000104157 |  | up |
| RAC1 |  | 2.548476768 |  | 0.000113762 |  | up |
| LRRK2 |  | 2.583792332 |  | 4.40804E-05 |  | up |
| PTPRC |  | 2.600964802 |  | 0.000146359 |  | up |
| SF3B1 |  | 2.727813787 |  | 1.01191E-05 |  | up |
| ATP5B |  | 2.78402453 |  | 6.07E-05 |  | up |
| STAT1 |  | 2.874379854 |  | 9.94825E-05 |  | up |
| HSPA8 |  | 2.890512558 |  | 5.59E-05 |  | up |
| UBB |  | 2.954022318 |  | 0.000340985 |  | up |
| PTEN |  | 3.31210705 |  | 6.16E-06 |  | up |
| UBA52 |  | 3.742899962 |  | 6.32328E-06 |  | up |
| ITGB1 |  | 3.832256417 |  | 0.000918943 |  | up |
| TLR4 |  | 3.999600962 |  | 2.87187E-05 |  | up |
| HIST2H2AC |  | 4.224546778 |  | 1.6707E-05 |  | up |
| CDC42 |  | 4.873470863 |  | 6.36941E-06 |  | up |

**Supplementary Table S7**

Immune cell abundance identifier (ImmuCellAI) fraction of infiltration of

immune cells in IgAV and normal group.

| Cell type | HD (%) |  | IgAV (%) |  | *p* value |
| --- | --- | --- | --- | --- | --- |
| InfiltrationScore | 72.75 |  | 78.15 |  | 0.35 |
| Th2 | 7.9 |  | 40.5 |  | 0.01 |
| Neutrophil | 16.85 |  | 33.05 |  | 0.07 |
| Monocyte | 7.05 |  | 26.15 |  | 0.01 |
| MAIT | 6.2 |  | 23.75 |  | 0.01 |
| Macrophage | 7.7 |  | 19.05 |  | 0.04 |
| Th1 | 5.65 |  | 16.3 |  | 0.11 |
| CD8_naive | 6.5 |  | 15.05 |  | 0.07 |
| Tfh | 2.15 |  | 11.35 |  | 0.04 |
| Th17 | 10.5 |  | 8.25 |  | 0.61 |
| Tr1 | 1.6 |  | 7 |  | 0.01 |
| iTreg | 5.8 |  | 6.45 |  | 0.33 |
| Gamma_delta | 4.3 |  | 6.45 |  | 0.11 |
| NK | 9.9 |  | 5.65 |  | 0.17 |
| NKT | 6.45 |  | 4.3 |  | 0.26 |
| Cytotoxic | 17.1 |  | 4 |  | 0.02 |
| Bcell | 15.45 |  | 3.7 |  | 0.02 |
| CD4_T | 8.65 |  | 2.35 |  | 0.11 |
| Central_memory | 3.85 |  | 2.15 |  | 0.33 |
| DC | 0 |  | 1.85 |  | 0.02 |
| Exhausted | 12.9 |  | 1.85 |  | 0.01 |
| CD8_T | 8.5 |  | 1.85 |  | 0.01 |
| CD4_naive | 7.5 |  | 0 |  | 0.01 |
| nTreg | 9.1 |  | 0 |  | 0.15 |
| Effector_memory | 5.75 |  | 0 |  | 0.01 |

**Supplementary Table S8**

Baseline demographics and clinical characteristics of patients for flow cytometry validation

| Characteristics | IgAVN  (n=30) | HD  (n=30) | *p* value |
| --- | --- | --- | --- |
| Age (year) | 10.7±1.8 | 9.1±2.5 | 0.139 |
| Females, n (%) | 14(46.67) | 17(56.67) | 0.447 |
| IgA (g/L) | 2.6±1.2 |  |  |
| IgG (g/L) | 10.7±2.3 |  |  |
| IgM (g/L) | 1.0±0.5 |  |  |
| CRP (mg/L) | 6.8±11.0 |  |  |
| ESR (mmol/L) | 16.5±14.5 |  |  |
