# Supplementary figures and images for: Identification of key genes and imbalance of immune cell infiltration in immunoglobulin A associated vasculitis nephritis by integrated bioinformatic analysis
Source: Front Immunol. 2023 Mar 21;14:1087293. doi: 10.3389/fimmu.2023.1087293 (PMC10070996; doi:10.3389/fimmu.2023.1087293)

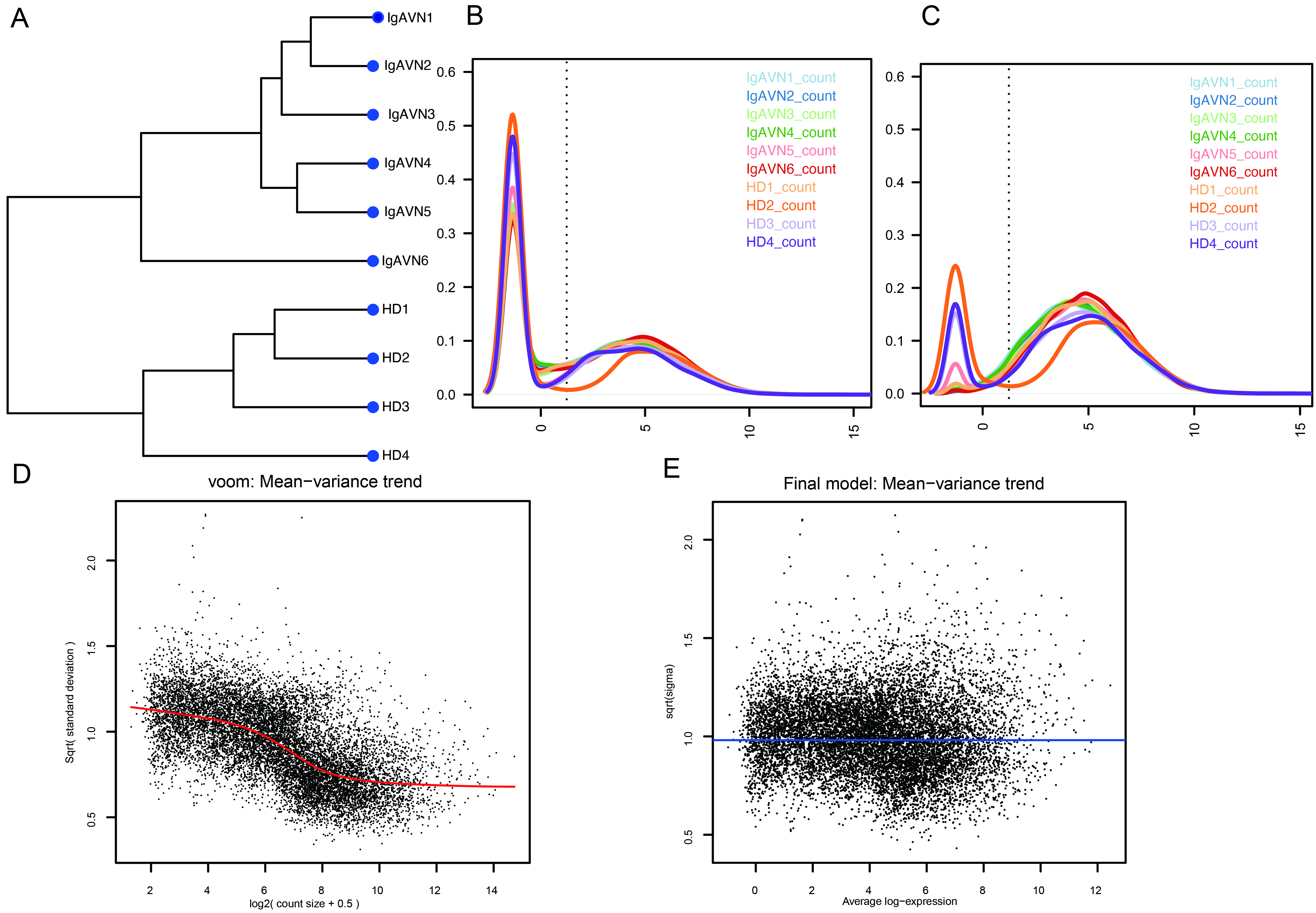

Supplement: Supplementary Figure 1 — Removing low expressed genes and normalization of gene expression for the GSE102114 dataset. (A) The hierarchical clustering of the samples from GSE102114 dataset. (B) The density plot of logCounts per Million (CPM) values for raw pre-filtered gene expression data. (C) The density plot of logCPM values after filtering low expressed genes. (D) The relationship between means and variances of each gene before voom. (E) The relationship between means and variances of each gene after voom. [file Image_1.tif]

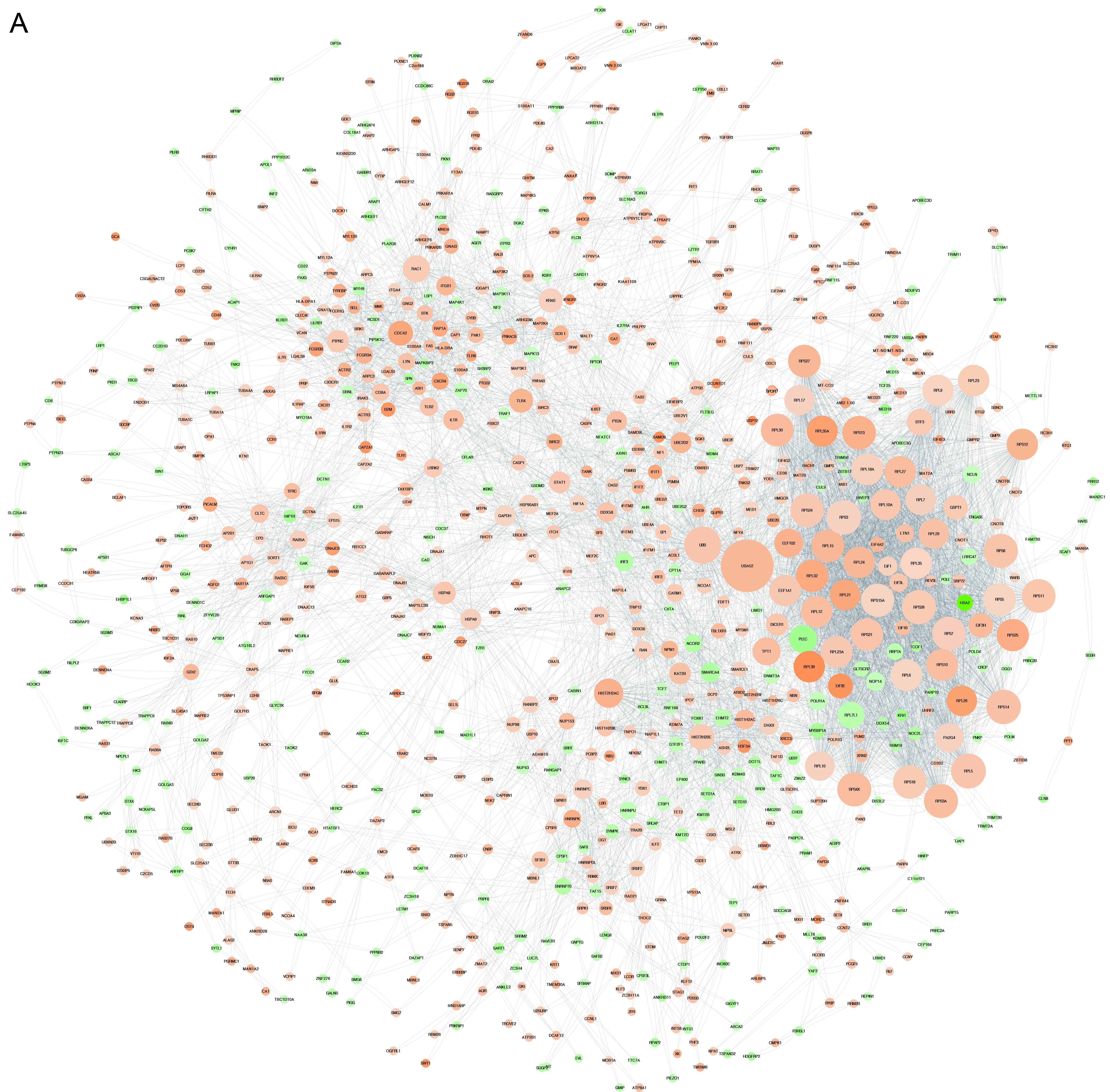

Supplement: Supplementary Figure 2 — Construction of the protein-protein interaction (PPI) network (A) The PPI network of DEGs, according to the STRING online database, was constructed by Cytoscape. [file Image_2.tif]

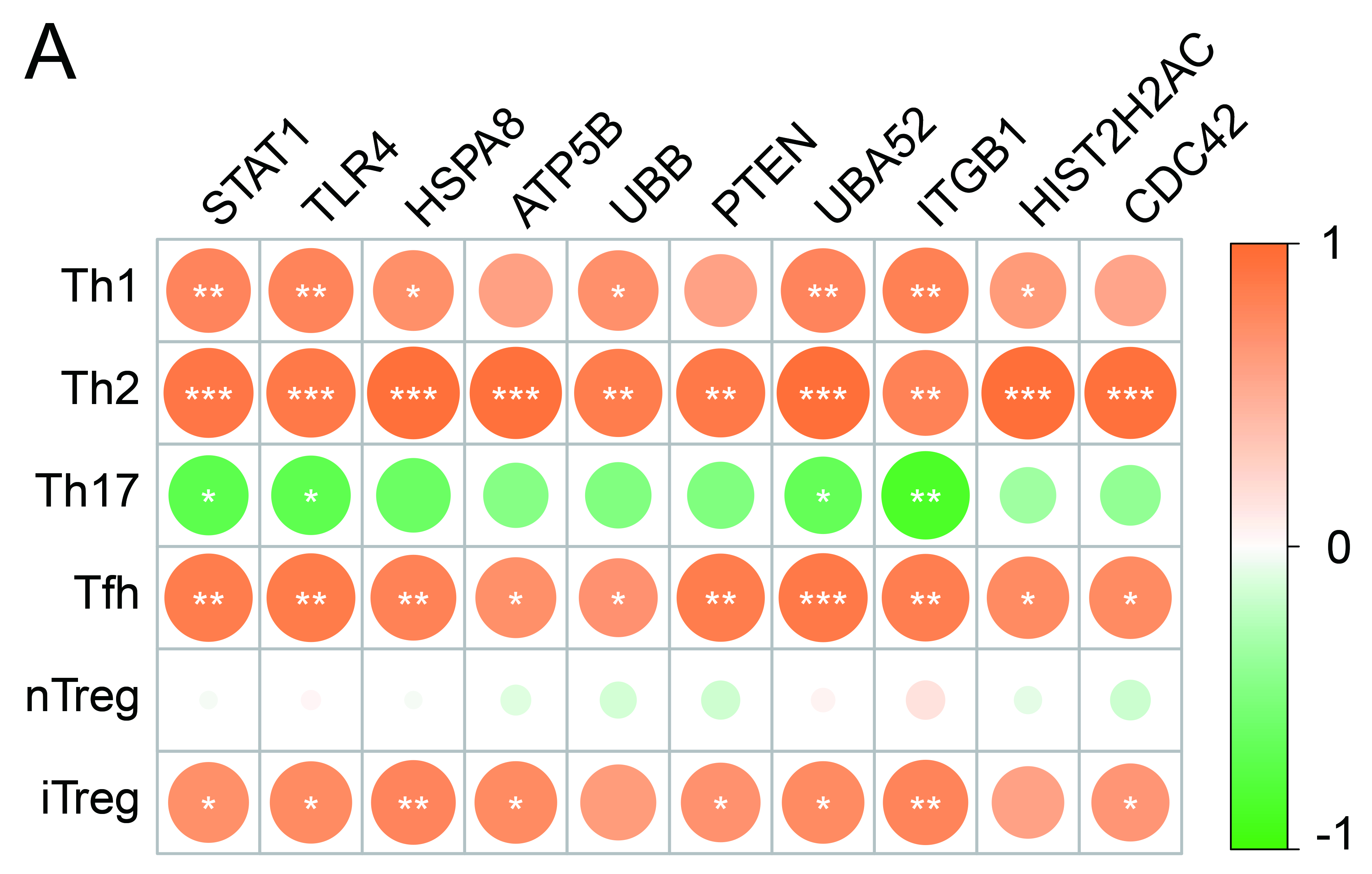

Supplement: Supplementary Figure 3 — Correlation analysis between 10 hub genes and subtypes of CD4+ T cell. The orange color represent positive correlation. The green color represent negative correlation. The size of the circle represent the degree of correlation ranging from 1 to -1. *P <0.05, **P <0.01, ***P <0.001. [file Image_3.tif]
